# Sensing red blood cell nano-mechanics: Toward a novel blood biomarker for Alzheimer’s disease

**DOI:** 10.3389/fnagi.2022.932354

**Published:** 2022-09-20

**Authors:** Matteo Nardini, Gabriele Ciasca, Alessandra Lauria, Cristina Rossi, Flavio Di Giacinto, Sabrina Romanò, Riccardo Di Santo, Massimiliano Papi, Valentina Palmieri, Giordano Perini, Umberto Basile, Francesca D. Alcaro, Enrico Di Stasio, Alessandra Bizzarro, Carlo Masullo, Marco De Spirito

**Affiliations:** ^1^Dipartimento di Neuroscienze, Sezione di Fisica, Università Cattolica del Sacro Cuore, Rome, Italy; ^2^Fondazione Policlinico Universitario Agostino Gemelli IRCCS, Rome, Italy; ^3^Unitá Operativa Complessa Neuroriabilitazione ad Alta Intensitá, Fondazione Policlinico Universitario A. Gemelli IRCCS, Rome, Italy; ^4^Department of Laboratory Diagnostic and Infectious Diseases, Fondazione Policlinico Universitario Agostino Gemelli IRCCS, Rome, Italy; ^5^Istituto dei Sistemi Complessi (ISC), Consiglio Nazionale delle Ricerche (CNR), Rome, Italy; ^6^Unitáă Operativa Complessa Continuità assistenziale, Fondazione Policlinico Universitario A. Gemelli IRCCS, Rome, Italy; ^7^Sezione di Neurologia, Dipartimento di Neuroscienze, Università Cattolica del Sacro Cuore, Rome, Italy

**Keywords:** Alzheimer’s disease, biomarker, liquid biopsy, AFM, mechanics, red blood cells

## Abstract

Red blood cells (RBCs) are characterized by a remarkable elasticity, which allows them to undergo very large deformation when passing through small vessels and capillaries. This extreme deformability is altered in various clinical conditions, suggesting that the analysis of red blood cell (RBC) mechanics has potential applications in the search for non-invasive and cost-effective blood biomarkers. Here, we provide a comparative study of the mechanical response of RBCs in patients with Alzheimer’s disease (AD) and healthy subjects. For this purpose, RBC viscoelastic response was investigated using atomic force microscopy (AFM) in the force spectroscopy mode. Two types of analyses were performed: (i) a conventional analysis of AFM force–distance (FD) curves, which allowed us to retrieve the apparent Young’s modulus, E; and (ii) a more in-depth analysis of time-dependent relaxation curves in the framework of the standard linear solid (SLS) model, which allowed us to estimate cell viscosity and elasticity, independently. Our data demonstrate that, while conventional analysis of AFM FD curves fails in distinguishing the two groups, the mechanical parameters obtained with the SLS model show a very good classification ability. The diagnostic performance of mechanical parameters was assessed using receiving operator characteristic (ROC) curves, showing very large areas under the curves (AUC) for selected biomarkers (AUC > 0.9). Taken all together, the data presented here demonstrate that RBC mechanics are significantly altered in AD, also highlighting the key role played by viscous forces. These RBC abnormalities in AD, which include both a modified elasticity and viscosity, could be considered a potential source of plasmatic biomarkers in the field of liquid biopsy to be used in combination with more established indicators of the pathology.

## Introduction

Currently, Alzheimer’s disease (AD) diagnostics rely on cognitive testing supported by additional biomarkers, which include cerebrospinal fluid Aβ42, total and phosphorylated full-length-tau or its truncated form, positron emission tomography (PET) of brain amyloid deposition and glucose metabolism, and brain atrophy on MRI (Cross et al., 2007; Battisti et al., 2012; Okamura et al., 2014; Brier et al., 2016; Florenzano et al., 2017; Wojsiat et al., 2017). Although considerable progress has been made in demonstrating how these biomarkers relate to the pathophysiology of AD (Jack et al., 2013), the search and validation of cost-effective and less invasive biomarkers, contributing to the early diagnosis of AD and the prediction of disease progression, are highly demanded (Snyder et al., 2014). In this context, blood biomarkers are specifically desirable because they reduce costs, and minimize the risk and the discomfort for the patients, thus stimulating the development of novel high-throughput screening methods.

Two classes of biomarkers can be distinguished in blood: circulating molecules and cell-based biomarkers (Wojsiat et al., 2017). The latter class has recently attracted a lot of attention, especially for what concerns red blood cells (RBCs), because of their easy and large accessibility. In this context, it has been demonstrated that 15% of RBCs in patients with AD showed an elongated shape, associated with the presence of alterations in the RBC membrane architecture (Mohanty et al., 2010). Moreover, a large body of evidence pointed out an association between RBCs and Aβ-peptides in patients with AD, which negatively affects cell integrity, functionality, and adhesion properties, thus contributing to cerebral hypoperfusion, favoring vascular damages, and eventually facilitating AD (van Oijen et al., 2006; Nakagawa et al., 2011; Kiko et al., 2012; Lucas and Rifkind, 2013). The association between Aβ and RBCs is not only involved in functional impairments of erythrocytes but also induces detectable shape and structural changes in the RBC morphology and membrane roughness, as recently demonstrated by Carelli-Alinovi et al. (2019). This experimental evidence highlights the occurrence of biochemical and morphological alterations of RBCs obtained from patients with AD in comparison with control subjects, suggesting that such alterations could be a potential source of blood biomarkers.

However, in the last two decades, it has been shown that both morphological and biochemical changes are deeply connected to a modification of the mechanical properties of cells and tissues, stimulating a significant research effort toward the identification and validation of novel mechanical biomarkers of pathologies (Suresh et al., 2005; Suresh, 2007; Cross et al., 2008; Shieh, 2011; Swaminathan et al., 2011; Ethier and Simmons, 2013; Choi et al., 2014; Van Zwieten et al., 2014; Minelli et al., 2017, 2018; Pichiecchio et al., 2018; Carelli-Alinovi et al., 2019). This is particularly true for RBCs for which mechanical deformability is a key characteristic. RBCs, indeed, need to undergo multiple deformations, when traveling through small blood vessels and organs (Dao et al., 2003; Ciasca et al., 2015). This remarkable deformability is closely related to RBC membrane structure, primarily consisting of a phospholipid bilayer with an underlying two-dimensional network of spectrin molecules. There is growing evidence that this extreme deformability is significantly altered in several pathological conditions, such as diabetes mellitus, essential hypertension, arteriosclerosis, and coronary artery, hereditary spherocytosis, thalassemia, G6PD deficiency, sickle cell disease, and that such alteration contributes to enhancing the flow resistance of blood (Barnes et al., 1977; Cooper et al., 1977; Brown et al., 2005; Lekka et al., 2005; Dulińska et al., 2006; Maciaszek and Lykotrafitis, 2011; Pretorius, 2013; Tomaiuolo, 2014; Ciasca et al., 2015). On the one hand, RBC modifications occur at the cellular level, on the other hand, they are closely related to changes in the molecular composition and organization of the cell that, in their turn, occur at the nanoscale level. This has made it necessary to develop quantitative tools able to probe RBC biomechanical changes at the nanometer and piconewton scales. In this context, atomic force microscopy (AFM) is an extremely powerful technique as it permits probing cells, tissues, and molecules at the nanoscale level in nearly physiological conditions (Cappella and Dietler, 1999; Kuznetsova et al., 2007; Montis et al., 2020; Ridolfi et al., 2020; Romanò et al., 2020; Di Santo et al., 2021). In this study, we investigate the viscoelastic properties of RBCs obtained from patients with AD, intending to search and validate possible blood biomarkers of the pathology that can be used for diagnostic applications as well as for therapy monitoring. For this purpose, we compared conventional AFM analysis with the Sneddon model with a more detailed analysis based on the application of the standard linear solid (SLS) model.

## Materials and methods

### Atomic force microscopy measurements: Patients’ recruitment, sample preparation, and data analysis

**FIGURE 1:**
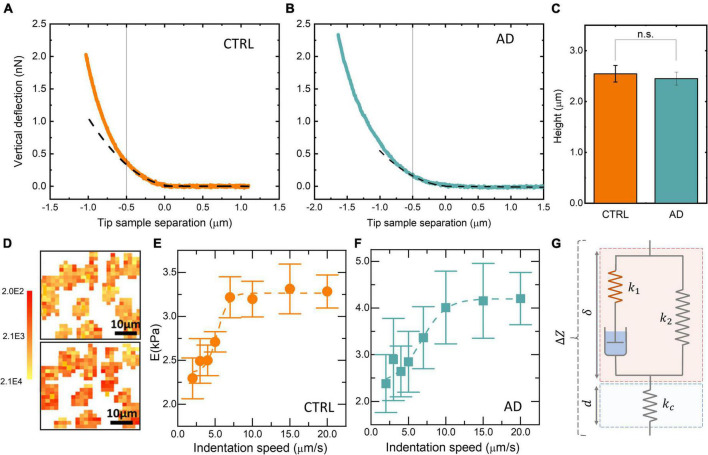
Representative force–distance (FD) approach curve acquired on a red blood cell (RBC) obtained from a control subject **(A)** and an Alzheimer’s disease (AD) patient **(B)**. Average RBC height in liquid environment **(C)**. Two representative RBC maps acquired at 20 μm/s (**D**, upper panel) and 5 μm/s (**D**, lower panel). Average Young’s modulus E as a function of the indentation speed for a control subject **(E)** and a patient with AD **(F)**. Schematic view of the standard linear solid (SLS) model **(G)**.

We observed a total of 53 blood samples from patients diagnosed with AD (26 subjects) and healthy controls (27 subjects). Patients with AD were selected by clinical and neuropsychological evaluation showing a diffuse cognitive impairment and were classified as probable AD according to the standardized clinical diagnostic criteria (McKhann et al., 2011). Patients with AD at the time of blood sampling were not treated with acetylcholinesterase (AchE) inhibitors or antiplatelet drugs. Prior measurements samples were centrifuged to separate serum from blood and red blood cells were deposited on a poly-L-lysine coated Petri dish. Force mapping measurements were performed at room temperature and in physiological solution (0.9% NaCl, Fresenius Kabi), using a JPK Nano Wizard II (JPK Instruments, Berlin, Germany) atomic force microscope equipped with silicon cantilevers with conical tip [MIKROMASCH CSC38 (Sofia, Bulgaria), nominal spring constant 0.03 N/m]. The cantilever spring constant was determined before each measurement by thermal calibration. We acquired a map of 64 force–distance (FD) curves and a map of 64 force–relaxation (FR) curves for each RBC for an average of 10 RBCs per sample. Each map covered the whole RBC surface and a portion of the unoccupied Petri surface. The latter region was eliminated before data analysis. Indentation curves were acquired using an indentation force of 2 nN at 5 μm/s indentation speed and were analyzed using the Sneddon model:


(1) $$F\,\left(\delta \right)=\frac{2\,E\,t\,a\,n\,\left(\alpha \right)}{\pi \,\left(1-\nu^{2}\right)}\,\delta^{2}$$

where *E* accounts for the apparent Young’s modulus, υ for the Poisson ratio, and δ for the indentation depth. The Poisson ratio was set at 0.5 to account for material incompressibility. Representative FD curves, together with the best fit of Eq. 1 to data, are shown in Figures 1A,B. The term apparent Young’s modulus (referred to as E in the following) accounts for the fact that, for sharp tips, E depends on both, the scanner velocity during indentation and the indentation depth. An indentation depth of 0.5 microns has been selected to analyze the data, as most of the measured FD curves displayed a parabolic behavior up to this threshold value, in agreement with Eq. 1. However, we note that this indentation depth exceeds the 10% threshold on the overall sample thickness (Figure 1C) and therefore, we might have induced an overestimation of the measured *E*-values, which could complicate the comparison with other AFM studies exploiting different experimental conditions or AFM set-ups. However, we believe that this overestimation is not affecting the comparison between the two groups, as the measured RBCs’ thickness is not significantly different in patients with AD and controls (Figure 1C).

**FIGURE 2:**
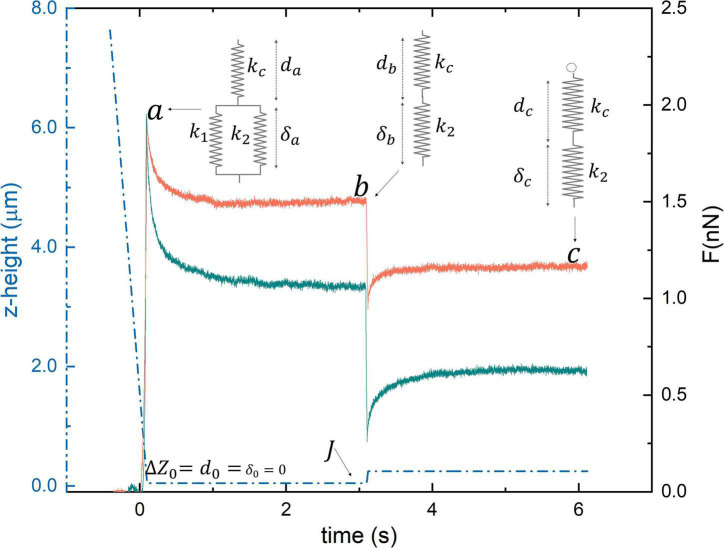
Two representative loading/unloading time-dependent relaxation curves acquired on a healthy (orange) and pathological (cyan) subject.

The behavior of Young’s modulus E as a function of the indentation speed in the range 1–20 μm/s was also investigated to assess the relevance of dissipative contributions in the determination of E (two representative curves are shown in Figures 1D–F). This analysis is extremely time-consuming and therefore it has been carried out on a reduced number of cells obtained from a small subset of the recruited subjects. For this purpose, 50 × 50 μm elasticity maps have been acquired (two representative images are shown in Figure 1D). The application of Eq. 1 relies on the assumption that the sample behaves like an elastic body. Biological samples rarely verify this assumption leading to well-known problems in the determination of E. To overcome this limitation, we acquired specifically designed time-dependent force–relaxation curves. These measurements were performed on a subset of the recruited subjects. Specifically, a total of 16 patients and 20 controls were subjected to this type of analysis. According to Rianna and Radmacher (2017), we studied cell mechanics with the SLS model, a theoretical framework that describes the sample as a linear combination of two elastic springs (*k*_1_ and *k*_2_) and a viscous damping element, *f* (Figure 1G). An additional elastic spring, *k_c_*, accounts for the cantilever contribution (Figure 1G). The following variables were defined: the cantilever position *Z*, deflection *d*, and the sample indentation δ (Figure 1G). It is worth stressing that δ cannot be measured directly, but it is obtained from the knowledge of Z and d. For these variables, the following relationship can be established: △*Z* = *d* + δ → δ = △Z−δ (Figure 1G). At the contact point, immediately before indenting the sample, we can set △*Z* = *d*_0_ = δ_0_ = 0 (Figure 2), as the cantilever is not deflected, the sample is not indented, and the *Z* = 0 position can be chosen arbitrarily (Figure 2). During the approach and indentation phase, we exploited a high indentation speed (35 μm/s) to avoid possible sample relaxation during indentation, before the target force is reached (point *a*, Figure 2). In this abrupt indentation phase, it can be assumed that the viscous dashpot has had no time to respond and, thus, the sample in the point *a* behaves like the parallel between *k*_1_ and *k*_2_. A dwell phase at constant height is then imposed, where the piezoelectric extension remains constant and the sample is allowed to relax (Figure 2). During this phase, $△Z=cost\to \dot{\delta }+-\dot{d}$ and the following time-dependent differential equation can be written:


(2) $$k_{c}\,d+\frac{f}{k_{1}}\,\left(k_{c}\,\dot{d}\right)=k_{2}\,\delta +f\,\frac{\left(k_{1}+k_{2}\right)}{k_{1}}\,\dot{\delta }$$

where k_*c*_ is the cantilever elastic constant, as measured with the thermal calibration method. In the constant height mode $(△Z=cost\to \dot{\delta }=-\dot{d)}$, Eq. 2 can be written as $d\,\left(t\right)=\frac{k_{2}}{\left(k_{2}+k_{c}\right)}\,△\,Z-\tau_{c}\,\dot{d}$, and solved as follows:


(3) $$d\,\left(\text{t}\right)-A\,e^{-\frac{t}{\tau_{c}}}+d_{b}$$

with $\tau_{c}=\frac{f\,\left(k_{1}+k_{2}+k_{c}\right)}{k_{1}\,\left(k_{c}+k_{2}\right)},$$A=\left(d_{a}-\frac{k_{2}}{\left(k_{c}+k_{2}\right)}\cdot △\,Z\right)$ and $d_{b}=\frac{k_{2}}{\left(k_{c}+k_{2}\right)}\cdot △\,Z$, where *d_a_* and *d_b_* are the cantilever deflections at points a and b (Figures 1F,G). The parameters *A*, τ_*c*_ and *d_b_* can be retrieved fitting the measured FR curves with Eq. 3 and used to calculate *k*_1_, *k*_2_, and *f*. Unfortunately, these quantities depend on ΔZ and *d_a_*, which are affected by a large experimental error in our set-up. To overcome this limitation, a height step is applied to the cantilever when the first relaxation is completed, retracting the tip of a known quantity J, as indicated in Figure 2. Being a pure elastic body, the cantilever instantly follows this variation, while the cell initially maintains its deformed state and then relaxes until a new equilibrium in point *c* is reached (Figures 1E–G). In the new equilibrium point *c*, the following equation can be written: *k*_*c*_*d*_*c*_ = *k*_2_ (△*Z*−*J*−*d*_*c*_), where *d_c_* is the measured deflection. Similarly, in point *a*, we can write *k*_*c*_*d*_*a*_ = (*k*_1_ + *k*_2_)(△*Z*−*d*_*a*_). Combining the latter five equations, we can get rid of ΔZ and *d_a_*, thus finding a mathematical expression for the SLS parameters (Eq. 4):


(4) $$\left\{ \begin{array}{c} k_{1}=\frac{A\,\left(k_{c}+k_{2}\right)\,k_{2}}{\left(k_{c}\,d_{b}-k_{2}\,A\right)}\\ k_{2}=\frac{k_{c}\,\left(d_{b}-d_{c}\right)}{\left(J+d_{c}-d_{b}\right)}\\ f=\tau_{c}\,\frac{k_{1}\,\left(k_{c}+k_{2}\right)}{\left(k_{1}+k_{2}+k_{c}\right)} \end{array}\right.$$

### Laboratory parameters

Clinical chemistry assay are highly automated and tests performed are closely monitored and quality controlled. The specimens are on serum or plasma analysis with techniques such as spectrophotometry and immunoassays to measure the concentration of substances (glucose, lipids, enzymes, electrolytes, hormones, proteins, and metabolic products) present in human blood. A complete blood count test measures several components and features of blood (amount of red blood cells, white blood cells, and platelets). The cytometric methods include cell size, cell count, and cell morphology.

### Statistical analysis

Statistical analyses were performed using the software package R (3.5.2 release) (R Development Core Team, 2016). Biomarkers were tested for normality by a visual inspection of the Q–Q plot followed by a Shapiro–Wilk test. It was found that selected biomarkers show some degree of deviation from normality; therefore, an unpaired two-samples Wilcoxon Test was used for group comparison. Analysis of covariance was used to account for covariates such as age (Table 1). The diagnostic accuracy of selected biomarkers in discriminating between the two groups was assessed by logistic regression and ROC curves. Logistic regression is executed using the function *glm* from the stats R package to extract probabilities from the fitted models, either with a single biomarker or with several biomarkers used in combination. A backward stepwise logistic regression was used to select, among biochemical and biomechanical parameters, the most effective subset of biomarkers for discriminating the two groups. For this purpose, Akaike’s Information Criterion (AIC) was exploited. ROC curves and AUC values were calculated using the R package *pROC*. Correlation between variables was evaluated by linear regression analysis and by calculating Spearman’s correlation coefficients. The strength of correlation was judged using correlation coefficients of >0.70 as strong correlation, 0.30–0.70 as moderate correlation, and <0.3 as weak correlation (Napodano et al., 2021).

**TABLE 1:** ANCOVA table for the variables k_1_, k_2_, and f reported in Figure 2.

	Df	Sum sq	Mean sq	*F*	*P*
***k*1**					
Age	1	3.43E-07	3.43E-07	3.766	0.061159
Group	1	1.41E-06	1.41E-06	15.477	0.000421
Residuals	32	2.92E-06	9.12E-08		
***k*2**					
Age	1	9.79E-07	9.79E-07	1.412	0.24351
Group	1	5.71E-06	5.71E-06	8.231	0.00724
Residuals	32	2.22E-05	6.94E-07		
***f***					
Age	1	1.01E-06	1.01E-06	6.253	0.017713
Group	1	2.49E-06	2.49E-06	15.445	0.000426
Residuals	32	5.15E-06	1.61E-07		

## Results

**FIGURE 3:**
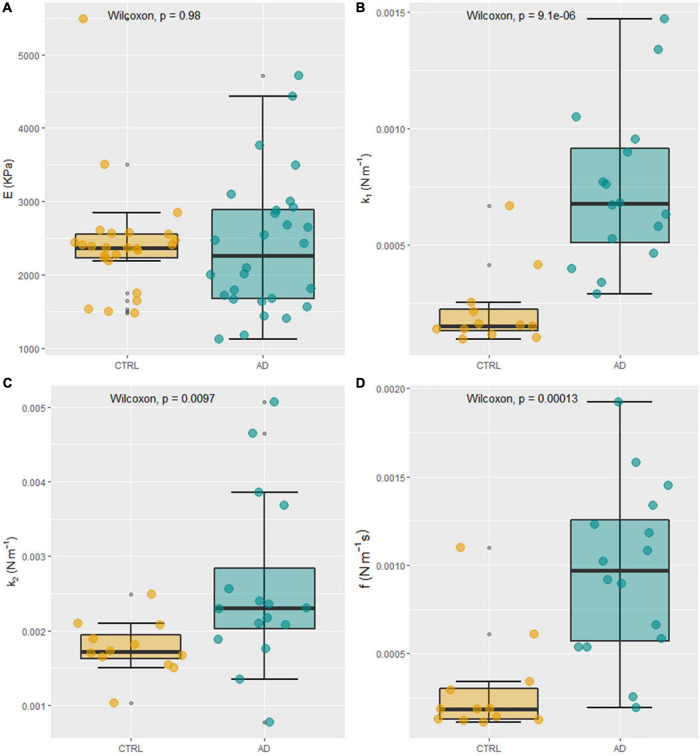
Box plot analysis of average *E*-values for control and pathological subjects **(A)**. Box plot analysis of mechanical parameters obtained from the standard linear solid (SLS) model **(B–D)**.

**FIGURE 4:**
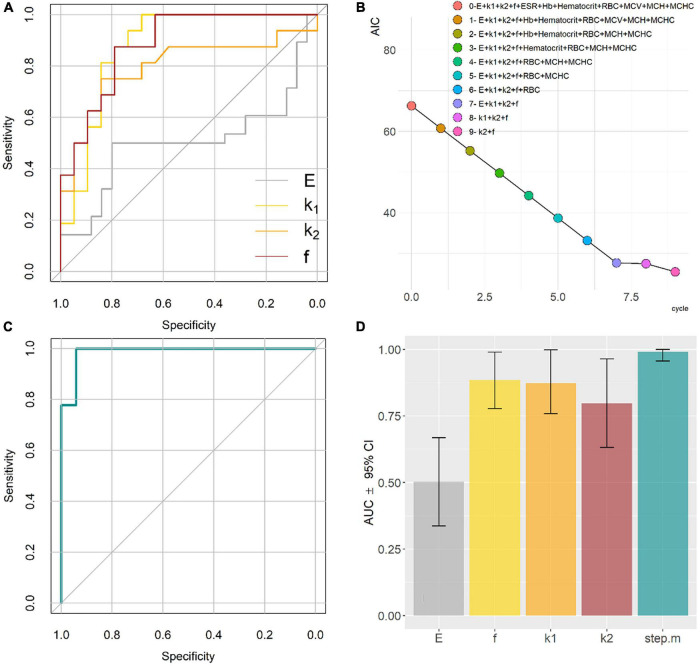
Receiving operator characteristic (ROC) curves for the four selected mechanical biomarkers **(A)**; Evolution of the Akaike’s Information Criterion (AIC) during a stepwise logistic regression performed on all the measured mechanical and biochemical parameters **(B)**; ROC curve calculated using the selected variables f and k2 **(C)**; Areas under the curves (AUC) values for the four mechanical parameters and the stepwise model **(D)**.

In Figures 1A,B, two representative FD curves acquired on an RBC obtained from a control subject and an AD patient are reported, respectively. A qualitative analysis of Figures 1A,B does not show marked differences between the two curves. However, a single-point measure cannot be considered representative of the biomechanical response of the whole cell. Therefore, we decided to probe the local response of each cell acquiring different FD curves at different positions over the cell surface. The Sneddon model (Eq. 1) was fitted to the experimental curves (black dashed lines in Figures 1A,B) to obtain the effective Young’s modulus, E. FD curves were fitted up to an indentation depth of 0.5 μm (black continuous lines in Figures 1A,B). This indentation range was chosen as the great majority of the analyzed curves showed the expected parabolic behavior up to this threshold. The experimental consequences of this choice are commented on in the Section “Discussion.” The representative fitting curves were prolonged up to 1 μm for visualization purposes. The mean *E*-value for each recruited subject was calculated by averaging the results obtained on all the measured cells. In Figure 1C, the average height of RBCs in liquid is shown for the two groups. Data are reported as mean ± SEM. No statistically significant differences are highlighted between the two groups. In most of the AFM studies exploiting Sneddon’s model or similar ones, the basic assumption is that the sample has a purely elastic behavior; hence dissipative forces are neglected, and Young’s modulus is treated as unaffected by probe dynamics. We checked for this assumption by measuring the average Young’s modulus E as a function of the cantilever speed during indentation in the range of 1–20 μm/s. The analysis was conducted over eight 50 × 50 μm maps. Two representative E maps acquired on a control subject at different indentation rates, *v* = 20 μm/s (Figure 1D, upper panel) and *v* = 5 μm/s (Figure 1D, lower panel) are reported. The same color scale was used for the two maps (Figure 1D, left). One can notice that the upper figure appears to be systematically brighter than the lower one in any location, pointing out a global stiffening of RBCs while increasing the indentation speed. In Figure 1E, a representative curve of the average Young’s modulus E as a function of the indentation rate is reported for a typical control subject. Data are shown as mean ± SEM. A sigmoidal curve was fitted to the data (dashed orange line). Increasing the scanner velocity, we observe a monotonous increase in E, which starts from approximately 2.3 kPa up to reaching a plateau value of 3.3 kPa at approximately 10 μm/s. The same analysis is carried out on a selected AD patient (Figure 1F), showing behavior that resembles the one reported in Figure 1E: we again observe a monotonous increase of E from approximately 2.3 kPa up to a plateau value of 4.25 kPa at approximately 15 μm/s. The reported sigmoidal behaviors are qualitatively in agreement with the SLS model, which is a mathematical way to evaluate the deformation properties of a sample as a linear combination of an elastic term (*k*_*1*_) and Maxwell’s arm, composed of an elastic element (*k*_*2*_) and a viscous dashpot (*f*) in series with each other (Figure 1G). It can be demonstrated that, according to this model, the sample reaction force at a very low indentation speed is dominated by elastic contributions. In the intermediate range, viscosity contributes to a non-linear increase of the reaction force, until it reaches a plateau at high speed. The qualitative agreement between the described trend and the data shown in Figures 1E,F demonstrate that RBCs do not have a purely elastic behavior. Conversely, their biomechanical response appears to be dependent on dissipative forces and viscous contributions (Leo et al., 2020). To measure viscous and elastic terms, separately, we acquired AFM time-dependent relaxation curves, as explained in the Section “Materials and methods.” For this purpose, we used a slightly modified version of the model proposed in Rianna and Radmacher (2016, 2017). In Figure 2, two representative FR curves are shown for a control subject and an AD patient, respectively. At variance with the typical FD curves (Figures 1A,B), where a clear difference between AD and control subjects was not observed, the two time-dependent curves appear different. To quantify this difference, we analyzed data using Eqs 2–4, which allowed us to retrieve the SLS parameters, namely, the damping element *f*, and the two elastic springs *k*_*1*_ and *k*_*2*_. Similarly to the conventional FD curves, a single-point measure is not representative of the biomechanical response of the whole cell; therefore, we acquired different time-dependent curves in different locations of the cell, and we averaged the results obtained on all the measured cells (on average 10 cells/subject). A box plot analysis of the average *E*-values calculated fitting Eq. 1 to conventional FD curves is shown in Figure 3A for control and pathological subjects. An unpaired two-samples Wilcoxon test was used to compare AD and control subjects, pointing out the absence of statistically significant differences between the two groups (*p* = 0.9878). In Figures 3B–D, a box plot analysis of the mechanical parameters obtained with the SLS model was shown. Additionally, a similar analysis is shown in Supplementary Figure 1 for τ_*c*_. Statistically significant *p*-values were obtained for all the SLS parameters (Figures 3B–D), namely, the damping element *f* (*p* = 9.1e-6) and the elastic terms *k*_*1*_ (*p* = 0.00097) and *k*_*2*_ (*p* = 0.0013). In this regard, a caveat is necessary as the two groups are not age-matched. Therefore, we performed an analysis of covariance (ANCOVA) analysis to decouple differences due to age. The results of this analysis show that the group membership is highly significant for all three variables, even if age is taken into account as a covariate. These findings suggest that, while the effective Young’s modulus E cannot be used to distinguish the two groups in our experimental conditions, SLS parameters are potentially useful for the development of novel mechanical biomarkers of the pathology. In the next section, we briefly commented on the possibility of using *E*-values obtained at higher indentation speeds to distinguish the two groups. ROC curves—a widely used statistical technique to evaluate and compare diagnostic tools (Hoo et al., 2017; Caputo et al., 2021; Di Santo et al., 2022)—were exploited to assess the performance of the four mechanical parameters (Figure 4A). ROC curves are two-dimensional graphs in which sensitivity, also referred to as the True Positive (TP) rate, is plotted against 1–specificity, which is the false positive (FP) rate (Metz, 1978). As specificity ranges between 0 and 1, ROC curves might be also reported in terms of sensitivity as a function of specificity, inverting the x-axis. In the present paper, the latter notation is used. The diagonal line represents the y = x bisector, which is the expected performance for a completely random classifier. ROC curves of the three SLS parameters rapidly increase for low values of the x-axis, showing that *f*, *k*_*1*_, and *k*_*2*_ are effective in distinguishing between the two groups. Conversely, Young’s modulus E fails in discriminating between control subjects and patients with AD. A widely used statistical metric for the quantitative evaluation of a ROC curve is the so-called area under the curve (AUC). By definition, AUC values lie in the range 0–1, where 1 corresponds to an ideal classifier and 0.5 to a completely random classifier; in general, the higher the AUC value, the better the classifier performance. As expected, large AUCs were measured for the three SLS parameters, namely, 0.88 (95% CI: 0.78–0.99) for *f*, 0.87 (95% CI: 0.76–0.99) for *k*_*1*_, and 0.80 (95% CI: 0.63–0.96) for *k*_*2*_. Young’s modulus E shows an AUC of 0.5 (95% CI: 0.34–0.67), which highlights its poor ability to discriminate between the two groups. To improve the effectiveness of the model shown in Figure 3B, we performed a stepwise logistic regression including all the measured biomechanical (E, *k*_*1*_, *k*_*2*_, *f*) parameters and several RBC indices routinely measured in blood tests, namely, erythrocyte sedimentation rate (ESR), RBC count, mean corpuscular volume (MCV), mean corpuscular hemoglobin (MCH), hemoglobin (Hb), and mean corpuscular hemoglobin concentration (MCHC). For this purpose, first, the complete model was obtained and then a stepwise backward selection was performed to highlight the most relevant subset of parameters that minimize AIC. Given the modest number of data points and to keep the model as simple as possible, we used the following information criterion that strongly penalizes large models with many parameters (n): $A\,I\,C=2\,k-2\,\ln\left(\tilde{L}\right)$, where *k* = *ln* (*n*) and $\tilde{L}$are the maximum value of the likelihood function for the model. The computed AIC value for each cycle of the stepwise regression procedure is shown in Figure 4B, showing the progressive removal of the less effective biomarkers. Notably, as shown in the figure, the procedure selects *f* and *k*_*2*_, while it discards all the biochemical parameters together with the effective Young’s modulus E and *k*_*1*_. In Figure 4C, we show the corresponding ROC curve for the selected model, which includes *f* and *k*_*2*_ and shows a very large AUC, 0.99 (95% CI: 0.96–1.00). The computed AUC values, together with the corresponding 95% CI, are summarized in Figure 4D.

**FIGURE 5:**
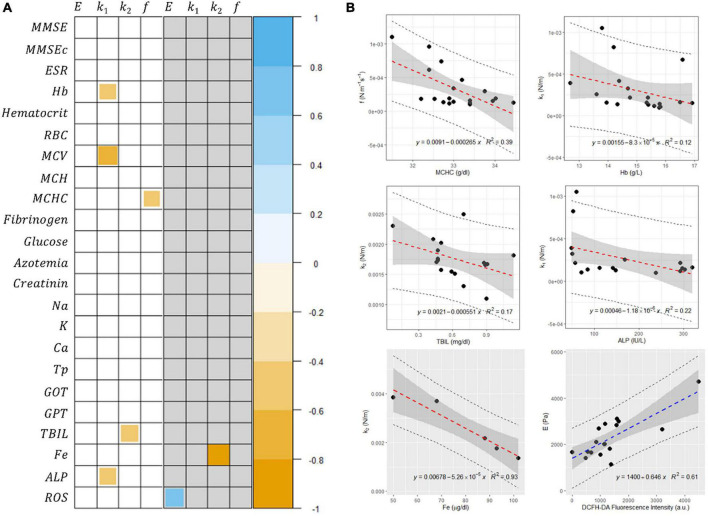
**(A)** Spearman correlation matrix between selected hematological parameters and mechanical biomarkers. Correlation maps are shown separately for control subjects (white background) and patients with Alzheimer’s disease (AD) (gray background). The two maps show only the statistically significant correlations (significance level 0.05). **(B)** Scatter plots showing the relationship between selected variables together with the corresponding linear regression analysis.

To investigate a more in-depth relationship between mechanical and biochemical parameters together with the results of neurological tests on patients with AD, we performed a correlational analysis between the two groups of variables. A biomarker correlation map is shown in Figure 5A for control subjects (white background) and patients with AD (gray background). The two maps report only the statistically significant correlations (significance level 0.05). In control subjects, a strong negative correlation ρ = −0.72 (*p* = 0.0008) was observed for the variables MCV and *k*_*1*_ and moderate negative correlations were observed for Hb/*k*_1_ ρ = −0.50 (*p* = 0.036), MCHC/*f ρ* = −0.50 (*p* = 0.034), TBIL/*k*_*2*_ ρ = −0.58 (*p* = 0.018), and ALP/*k*_*1*_ ρ = −0.55 (*p* = 0.028). In patients with AD, a moderate positive correlation of ρ = 0.66 (*p* = 0.00018) is observed for the concentration of reactive oxygen species (ROS) and E, and a strong negative correlation of ρ = −1.0 (*p* = 0.0072) was observed for Fe/*k*_*2*_. No other relevant correlations are shown. For the sake of completeness, in Figure 5B, we report the corresponding scatterplots, which were analyzed according to a linear model (this analysis was conducted only on the datasets which did not show marked deviation from normality).

## Discussion

In the last decades, a large body of evidence has emerged that—along with biochemical and genetic cues—mechanical stimuli are critical regulators in human physiology, as well as fundamental players in the onset and the progression of many pathological states (Kumar and Weaver, 2009; Lu et al., 2011; Van Zwieten et al., 2014; Ciasca et al., 2016a,^2019^; Vielmuth et al., 2016; Weaver, 2017; Perini et al., 2019; De-Giorgio et al., 2020; Di Giacinto et al., 2020; Mazzini et al., 2020). A wide range of diseases that bring patients to the doctor’s office can be, indeed, associated with significant alterations in the mechanical properties of cells, tissues, and organs. Mechanical deformability is also a major characteristic of RBCs, as they need to undergo large deformations when passing through small vessels and capillaries. Such extreme deformability is deeply altered in pathological conditions and inflammatory diseases (MacIaszek et al., 2011; Buys et al., 2013; Pretorius, 2013; Pretorius and Kell, 2014; Pretorius et al., 2015). In this context, the quantitative analysis of the biomechanical response of RBCs has potentially wide applications in diagnostics.

Here we presented a comparative AFM study of the mechanical properties of RBCs extracted from patients with AD and healthy control subjects. The study is aimed to identify possible mechanical biomarkers of the pathology directly from the blood, which could be exploited for diagnostic purposes and therapy monitoring.

For this purpose, two types of analyses were exploited. First, we performed a conventional analysis of AFM FD curves with the Sneddon model, which allowed us to retrieve the apparent Young’s modulus E of cells. E is one of the most widely used parameters to probe RBC stiffness in different pathological conditions. However, in this case, no statistically significant differences were found between the two groups (Figure 3A), as further confirmed by a ROC curve analysis (Figure 4A), showing that E has extremely poor classification abilities. This finding is in close agreement with the results of Bester et al. (2013), which first compared the biomechanical properties of RBCs extracted from control subjects and patients with AD, showing no significant differences among the elastic modulus values in the two groups (Ethier and Simmons, 2013). Interestingly, they found a significant increase in Young’s modulus of hyperferritinemic AD subjects compared to healthy and normoferritinemic AD ones, providing further evidence of the strong relation between AD, and ferritin levels and its metal content (De Sole et al., 2013). Many studies investigating the biomechanical properties of RBC rely only on the analysis of conventional FD curves with the Hertz model, the Sneddon model, or similar ones. In this regard, a caveat is necessary. These analyses implicitly assume that the sample behaves like an elastic body. This assumption cannot be considered strictly valid in our case, as shown in Figures 1D–F, where the apparent Young’s modulus E shows a sigmoidal dependence on the indentation speed. The analysis is carried out on two representative subjects randomly selected within the recruited population. Therefore, the comparison between the two curves cannot be used to draw a statistically relevant conclusion on the mechanical behavior of RBCs in the two groups. Nonetheless, this comparison is interesting as it suggests that the effective Young’s modulus E measured at 20 μm/s might be more effective in highlighting differences between the two groups, a hypothesis that deserves a more in-depth investigation in a dedicated paper. The E behavior shown in Figures 1D–F indicates that viscous forces contribute relevantly to RBC biomechanics and, thus, need to be included in the analysis of force spectroscopy measurements. Since the loading rate is not constant during the acquisition of FD curves on a soft sample, due to the change in the tip-sample contact area, extending the Sneddon model (or similar ones) to effectively include viscous effects is a hard and non-trivial task. An alternative way of estimating the contribution of dissipative forces in AFM experiments is to measure the percentage of energy dissipated during indentation, also referred to as hysteresis (H). Unfortunately, H does not provide a quantitative measure of sample viscosity, but rather a semi-quantitative estimation of dissipative contributions (Suresh, 2007; Cross et al., 2008; Ciasca et al., 2016b; Kulkarni et al., 2019).

To overcome these limitations and to measure the viscoelastic properties of cells and polymeric materials with AFM, several approaches have been proposed and tested (Ngan et al., 2005; Passeri et al., 2009; Moreno-Flores et al., 2010; Cartagena and Raman, 2014; Rianna and Radmacher, 2016, 2017; Heydarian et al., 2020; Zhang et al., 2020). In the present paper, we employed a slightly modified version of a model developed by Rianna and Radmacher (2016, 2017), as discussed in the Section “Materials and methods”. In their paper, the authors designed a specific sequence of time-dependent force–relaxation curves, which easily allows decoupling of viscous and elastic contributions in the framework of the SLS model. Interestingly, and at variance with Young’s modulus E, a statistically significant difference between patients with AD and control subjects was found for all the biomechanical parameters obtained with the SLS models (Figures 3B–D). Very often RBCs extracted from pathological subjects are reported to be simply stiffer than the ones obtained from healthy controls, based on Young’s modulus value obtained from the analysis of FD curves with Hertz’s model. This widely observed effect is likely to be due, not only to a change in RBC elastic properties—mainly associated with cytoskeletal alterations—but also to a change in cell viscosity that can be ascribed, among other factors, to the plasma membrane or hemoglobin modifications. These two effects can contribute in a complex fashion to the measured *E*-values obtained from FD curves, in some cases producing a detectable difference between the apparent cell stiffness in healthy and pathological erythrocytes, in some other cases—this one included—masking the actual impairment of cell mechanical properties. In the latter case, decoupling viscous and elastic terms is an effective strategy in the search for novel mechanical biomarkers of the pathology, as confirmed by the large AUC values obtained for the SLS parameters in Figure 4D.

This paper fits in the research areas related to the search and the validation of novel cell-based plasma biomarkers, which are highly desirable because of the reduced discomfort for patients, the generally low cost, and scalability to large screening for early diagnosis and prevention. At present, a wide panel of hematological and biochemical markers is routinely measured in diagnostics. To investigate more in-depth the relationship between the mechanical parameters presented in this paper and more established clinical parameters, we performed a correlation analysis between these two classes of blood indicators. Interestingly, few correlations were found between biochemical and mechanical parameters in the pathological group, suggesting that RBC biomechanics can be considered as a rather independent source of clinical information and, thus, can be used in combination with more established blood markers to get a comprehensive view of the patient’s clinical conditions. A second notable result is the presence of a moderate correlation between selected mechanical parameters and RBC indices, including MCV, MCH, and MCHC in the control group. Similar correlations were recently observed in the paper of Von Tempelhoff et al. (2016), studying a group of apparently healthy women. Our results show that the damping parameter *f* is significantly correlated only with the mean corpuscular hemoglobin concentration in healthy subjects. This result was expected as the hemoglobin concentration and its biochemical properties primarily determine intracellular fluid viscosity (Connes et al., 2014). Interestingly, we also detected the presence of moderate correlation between selected mechanical and hepatic parameters in the control group, namely, alkaline phosphatase (ALP) and total bilirubin. This result is particularly interesting and deserves a more in-depth study as it strengthens the link between the presence of a possible alteration in hepatic microcirculation and the impairment of the RBC mechanical properties.

Ultimately, we investigated the presence of correlations between the investigated mechanical parameters and the results of the Mini-Mental State Examination (MMSE), a neurological test extensively used in clinical and research settings to measure the cognitive impairment of patients (Tombaugh and McIntyre, 1992). This investigation aimed to assess whether the mechanical parameters could be used as a quantitative measure of the progression of the pathology. The MMSE corrected for patients’ age (MMSEc) was also investigated for the same reason. Unfortunately, none of the studied mechanical parameters shows any significant correlation with the MMSE score, indicating that RBC biomechanics as measured in this setting cannot be used to develop a quantitative biomarker of disease progression, an issue that can be regarded as a limitation hindering the applicability of the investigated markers. Moreover, it is also important to stress further limitations of the proposed technique. First, it is well-known that RBC nano-mechanics is highly sensitive to the inflammatory environment, which characterizes many clinical conditions other than AD, e.g., diabetes (Pretorius and Kell, 2014). Therefore, RBC mechanical properties cannot be considered specific markers of AD, but rather additional diagnostic parameters that have to be used in combination with more established clinical indicators to improve diagnostic specificity and sensitivity. Additionally, AFM is a time-consuming technique in terms of measurement time and data analysis and often requires specialized personnel with a physical background. Fortunately, these limitations can be overcome by using high-speed (HS-) AFM and machine learning for data analysis (Nievergelt et al., 2015; Wang et al., 2016; Dufrêne et al., 2017; Sokolov et al., 2018; Casuso et al., 2020; Dokukin and Dokukina, 2020; Ridolfi et al., 2020; Chandrashekar et al., 2022; Nguyen and Liu, 2022). To conclude, a further limitation of our work consists in the use of single exponential decays for fitting time-dependent curves such as those reported in Figure 2. We adopted this choice to keep the model as simple as possible so that data could be fitted automatically with homemade software, but a fitting function composed of multiple exponential decays (≥2) would be more appropriate than a single exponential decay. Therefore, similar to *E*-values, the SLS parameters here presented should be considered effective parameters, and special care must be paid when comparing their absolute value with similar values obtained with more complex models. Nevertheless, we believe that the mentioned simplification is not negatively affecting the diagnostic potential of our quantitative markers, as far as the same protocol is used for all the recruited subjects, irrespectively of the group membership.

## Conclusion

In order to pass through the microcirculation, RBCs need to undergo extensive deformations and recover their original shape. On the one hand, this extreme deformability is altered by various pathological conditions; on the other hand, an altered RBC deformability can have major effects on blood flow, leading to pathological implications. In this scenario, the investigation of the viscoelastic response of RBCs by AFM is highly effective to unveil subtle alterations of their deformability under pathological conditions. By using AFM in the force spectroscopy mode, we compared the biomechanical properties of RBC obtained from healthy donors and patients with AD, to search for novel blood biomarkers of the pathology. Our results show that Young’s modulus E, obtained with a conventional analysis of FD curves, fails in discriminating between control subjects and patients with AD. This is likely since this analysis relies on the assumption that the investigated cell has a purely elastic behavior. Such an assumption is rarely verified in soft and biological samples and, very often, the interplay of elastic and viscous contributions affects the determination of E in a complex fashion. To overcome this limitation, we applied a more in-depth analysis, which combines time-dependent force–relaxation curves with the SLS model, a general theoretical framework that allows describing the sample as a linear combination of elastic and viscous terms. Interestingly, the SLS parameters, namely, the damping term f and the elastic springs k_1_ and k_2_, are significantly different in the two classes of subjects, as further confirmed by the corresponding ROC curves with very large AUC values. A correlational analysis between mechanical parameters and biochemical ones was also performed. This analysis showed the presence of few mild correlations between the two classes of parameters, pointing out that RBC biomechanics is a potentially independent source of valuable clinical information. Taken all together, our results highlight the presence of significant abnormalities in the biomechanics of RBCs obtained from patients with AD, whose detection and quantification can positively influence the search for novel blood biomarkers of AD useful for diagnosis and therapy monitoring.

## Data availability statement

The data are available from the authors upon reasonable request.

## Ethics statement

The studies involving human participants were reviewed and approved by Ethical Committee of Università Cattolica del Sacro Cuore (protocol code no. 22237/19). The patients/participants provided their written informed consent to participate in this study.

## Author contributions

All authors have contributed substantially to the manuscript and have read and agreed to the published version of the manuscript.

## Supplementary material

Supplementary Figure 1 Box plot analysis of τ_*c*_ in the two groups.
