# Supplementary figures and images for: Sensing red blood cell nano-mechanics: Toward a novel blood biomarker for Alzheimer’s disease
Source: Front Aging Neurosci. 2022 Sep 20;14:932354. doi: 10.3389/fnagi.2022.932354 (PMC9530048; doi:10.3389/fnagi.2022.932354)

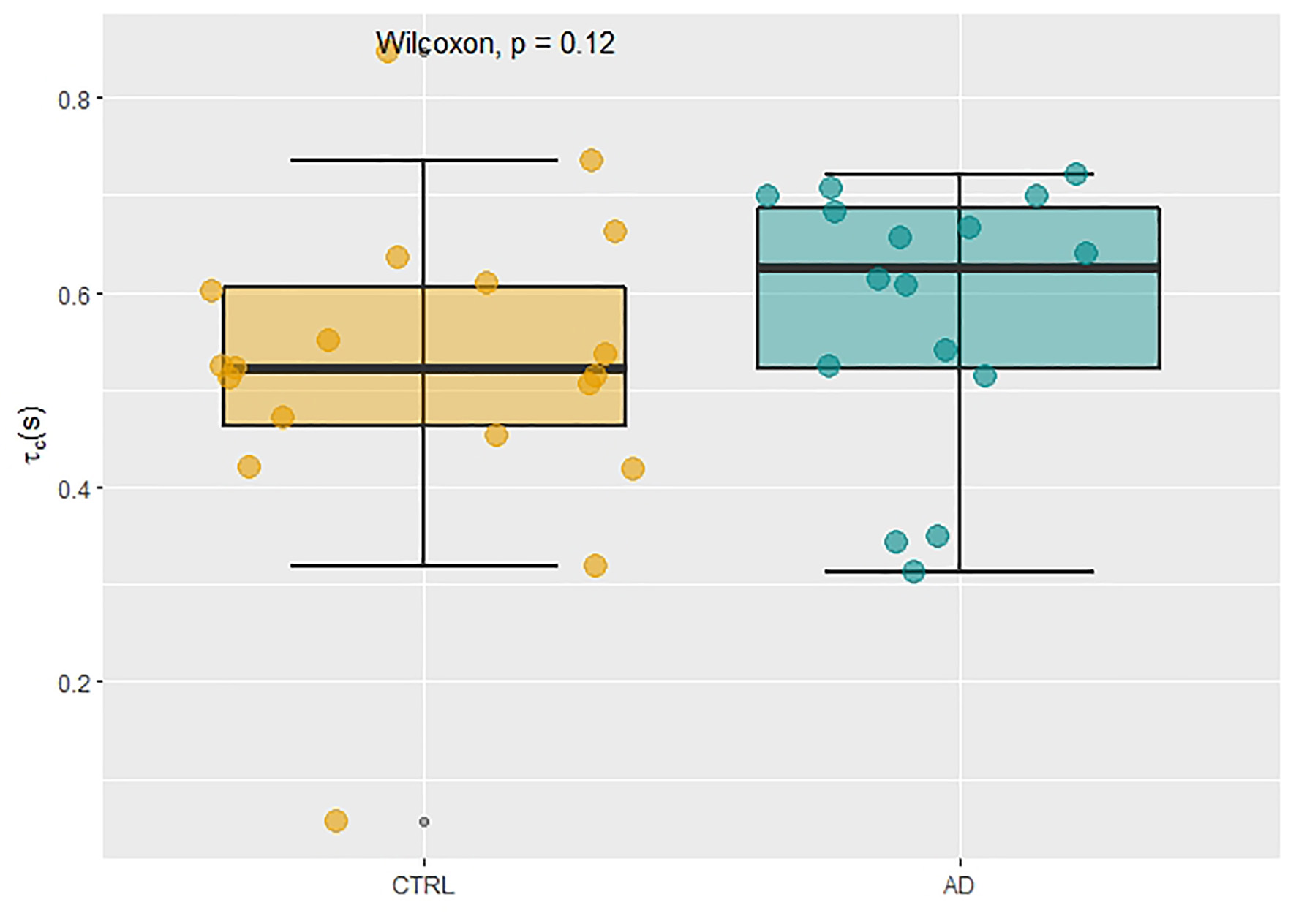

Supplement: Supplementary Figure 1 — Box plot analysis of τc in the two groups. [file Image_1.JPEG]
